# Perceptions and experiences of Korean American older adults with companion robots through long-term use: a comparative analysis of robot retention vs. return

**DOI:** 10.3389/fpubh.2024.1424123

**Published:** 2024-11-18

**Authors:** Othelia EunKyoung Lee, Ji-Chan Yun, Do-Hyung Park

**Affiliations:** ^1^School of Social Work, University of North Carolina Charlotte, Charlotte, NC, United States; ^2^Graduate School of Business IT, Kookmin University, Seoul, Republic of Korea

**Keywords:** companion robots, socially assistive robot, Korean Americans, older adults, user experiences, technology usability

## Abstract

To date, limited research has been conducted on technology use among socially marginalized groups, such as older immigrants who may have limited digital literacy. This pilot study aims to explore Korean American older adults’ perceptions and experiences with a companion version of the social robot, Hyodol. We hypothesize that the Hyodol robot’s social presence may facilitate technology use among this sample. To test this hypothesis, we invited 35 Korean American older adults to interact with Hyodol SAR over a four-month period. This extended engagement allowed us to investigate the underlying factors and dimensions shaping users’ perceptions and experiences. We assessed perceptions through measures of robotic attitudes and usability, while user experiences were evaluated using overall assessment questions and behavioral indicators, such as instances where participants showed the robot to others. We conducted a comparative analysis between participants who chose to keep the robot (“Keepers”) and those who opted to return it (“Returners”), providing insights into how each group utilized and interpreted the robots. Additionally, we examined the reasons Returners decided to not to retain the robot, aiming to identify barriers to acceptance and engagement. Our results indicated that participants’ experiences of warmth and competence while interacting with the Hyodol robots did not significantly differ between the Keepers and Returners. However, distinct patterns emerged in their utilization and interpretation of the robot; the 24 Keepers demonstrated a more intimate level of engagement compared to the 11 Returners. In an era characterized by the growing integration of AI in human care, our findings suggest that social presence became valuable concepts for developing robot companions to enhance their effectiveness.

## 1 Introduction

Social isolation refers to the objective condition of having limited social interactions or minimal contact with others (1). This measurable state occurs when individuals have few or no regular social connections, which can result in reduced social support and engagement (2, 3). In contrast, loneliness in older adults is defined as the subjective experience of emotional disconnection or the absence of meaningful social relationships, independent of the actual frequency of social contact (4). It represents an individual’s perception of being alone, even when in the presence of others (5, 6). Both loneliness, as an emotional response, and social isolation, as a situational or structural condition, have significant implications for the health and well-being of older immigrants.

Effective use of technology has been suggested as strategies to mitigate loneliness and social isolation among community dwelling older adults (7, 8). When adopting a novel technology users weigh the perceived benefits and ease of use against any potential drawbacks or difficulties (9). Older users are more likely to adopt technology when they perceive it as useful and easy to use, leading to a positive attitude and a higher intention to use, ultimately resulting in the technology’s actual use (10, 11). Considering the differential degree of technology acceptance, more research is needed to investigate underlying factors to motivate or discourage technology acceptance among older adults.

In recent years, research has increasingly focused on the impact of socially assistive robots (SARs) on older adults. Many studies have found that users perceive these robots, regardless of type and primary function, as more beneficial when they perform multiple tasks, such as assisting with daily activities, providing medication reminders, supporting cognitive functions, and encouraging engagement in various activities (12, 48). Features such as conversational interaction and reciprocal affection, particularly in companion robots were highlighted as desirable by users (13, 14). Additionally, smaller-sized robots were preferred, as they were perceived to be easier to work with and better suited to integrate into users’ daily routines (15).

The evaluation of the actual usage and usability of novel technologies such as SARs should be based on the experiences of users who have adapted to them over the long term. Further research is needed to evaluate users’ perceptions of usefulness and satisfaction with these robots over time, as their needs evolve with aging and changes in physical and cognitive abilities. Such insights will be crucial for developing effective robots that support aging-in-place. To date, limited study has been conducted to examine technology acceptance among socially invisible groups such as older immigrants who may have possess limited digital literacy (16).

Most Korean American older adults experienced the Korean War during childhood and undertook transcontinental immigration in adulthood (17). Due to their relatively recent immigration, they are uniquely affected by migratory grief, acculturation stress, race-based discrimination, and limited English proficiency (18). Generational and cultural differences in values, expectations, and language can cause tensions between Korean American older adults and their American-born children or grandchildren, further exacerbating emotional strain and poor quality of life (19). Some may confront financial hardships, which in turn can constrain their ability to partake in social engagements or access resources capable of mitigating their isolation (20).

Moreover, within immigrant communities, there is often a prevailing stigma linked to acknowledging feelings of loneliness or seeking assistance for mental health concerns outside of family (2). In many situations culturally relevant services are not within their reach geographically. Therefore, the confluence of language barriers, health challenges, stigma, digital illiteracy, and a dearth of culturally responsive services can contribute to social isolation, loneliness, depression, and healthcare disparities among Korean American older adults (5). During the peak of the COVID-19 pandemic, Perry et al. (21) undertook a study to assess the healthcare needs of a diverse urban older population. Results indicated that just over a quarter of this demographic experienced unmet healthcare needs. Notably, Asian Americans stood out, reporting the highest rate at 36% for unaddressed healthcare needs.

Hyodol robots, designed to cater to the needs of older adults, has been distributed to over 10,000 low-income older adults living alone in various counties across South Korea (22). Studies conducted in Korea have demonstrated a significant reduction in depressive symptoms among solo-living, low-income older adults who interact with companion version of SAR named Hyodol (23). Furthermore, Lee et al. (24) demonstrated a high level of adoptability for robots among those with limited education residing in rural areas during the COVID-19 pandemic. Based on these previous studies in South Korea we questioned whether Hyodol robot may also have potential as an assistive resource for older Korean American immigrants. Empirical validation of this social companion robot may demonstrate its potential as an effective solution for socially isolated older immigrants.

### 1.1 Social presence theory

This study was guided by Social Presence Theory, a framework used to understand the ways in which people perceive and interact with other entities in a mediated environment, such as robots (25). Social presence refers to the degree to which a person feels or perceives the presence of robots as if it were a real, socially meaningful being. It is essentially the sense of “being there” with another entity in a communication or interaction setting (26). In human-robot interactions, social presence theory suggests that the degree to which a robot is perceived as “socially present” can influence how users interact with it (10). Users experience varying degrees of social presence in different types of mediated interactions, depending on how much they feel as though they are interacting with another human being.

This perception can be influenced by a range of factors, including the robot’s appearance, behavior, and communication style. For example, a robot that is designed to look and act like a human may be perceived as more socially present than a robot that looks like a machine, because they trigger anthropomorphic responses in humans (27). Robots that exhibit human-like gestures, facial expressions, speech, and body language are more likely to be perceived as socially present.

The individual’s own disposition, attitudes, and expectations play a crucial role in perceiving social presence (28). Some people may be more willing to accept robots as social beings, while others may resist such perceptions. We hypothesized that high social presence could lead to increased trust in robots, making users more willing to cooperate and engage with them in various tasks. When robots exhibit social presence, users may emotionally engage with them, which can be beneficial in applications such as therapy or education. People may be more likely to follow instructions or guidance from robots perceived as socially present. However, high social presence can lead to ethical dilemmas, as users may attribute intentions and emotions to robots that they do not actually possess (7).

Additionally, culture profoundly influences technology use among Asian American older adults, affecting everything from adoption rates and usage patterns to preferences and trust levels (29). Hence, understanding these cultural factors is crucial for designing and implementing technologies that meet the unique needs of diverse populations. To illustrate, their values on family and community may lead to a preference for technologies that facilitate communication and emotional support. To enhance the adoption of technology, it is essential to provide interfaces and support in users’ native languages.

### 1.2 The present study

Korean American older adults invited to interact with Hyodol robots for the duration of 4 months. In our pilot study, we utilized a one-group pre- and post-test design to evaluate changes between baseline and follow-up. Post-test results indicated improvements in medication adherence and reductions in depressive symptoms (24). The current study aims to further explore users’ perceptions and experiences with the companion version of the SAR as a complementary assistive resource, focusing on how users perceive the robot across various dimensions during long-term use. These perceptions shape users’ interactions with the robot, leading to accumulated experiences that ultimately influence their decision to either continue using the robot or return it. We hypothesize that there will be notable differences in the experiences of those who choose to keep Hyodol compared to those who decide to return it, and this study seeks to investigate these differences. The following research questions were investigated in this study:

1. *What are the underlying factors and dimensions that can explain users’ perceptions of the robot after long-term use?*
2. *What are the differences in experiential evaluations between users who adopted the robot and those who returned it after long-term use?*

## 2 Methods

### 2.1 Participants

The study recruited Korean American immigrant older adults living in a metropolitan area within the northeastern United States. These individuals were recruited from a large community center primarily serving low-income Asian American older adults. To be eligible for participation, individuals had to be Korean immigrants aged 65 or older who could engage in verbal conversations in Korean. Preference was given to individuals who were low-income, living alone, and did not possess a pet. Each participant granted written informed consent, outlining the safeguarding of their health and privacy information. The research protocol was approved by the Institutional Review Board at the participating university (UNC Charlotte IRB-22-1137).

### 2.2 Companion robot intervention

**Figure 1:**
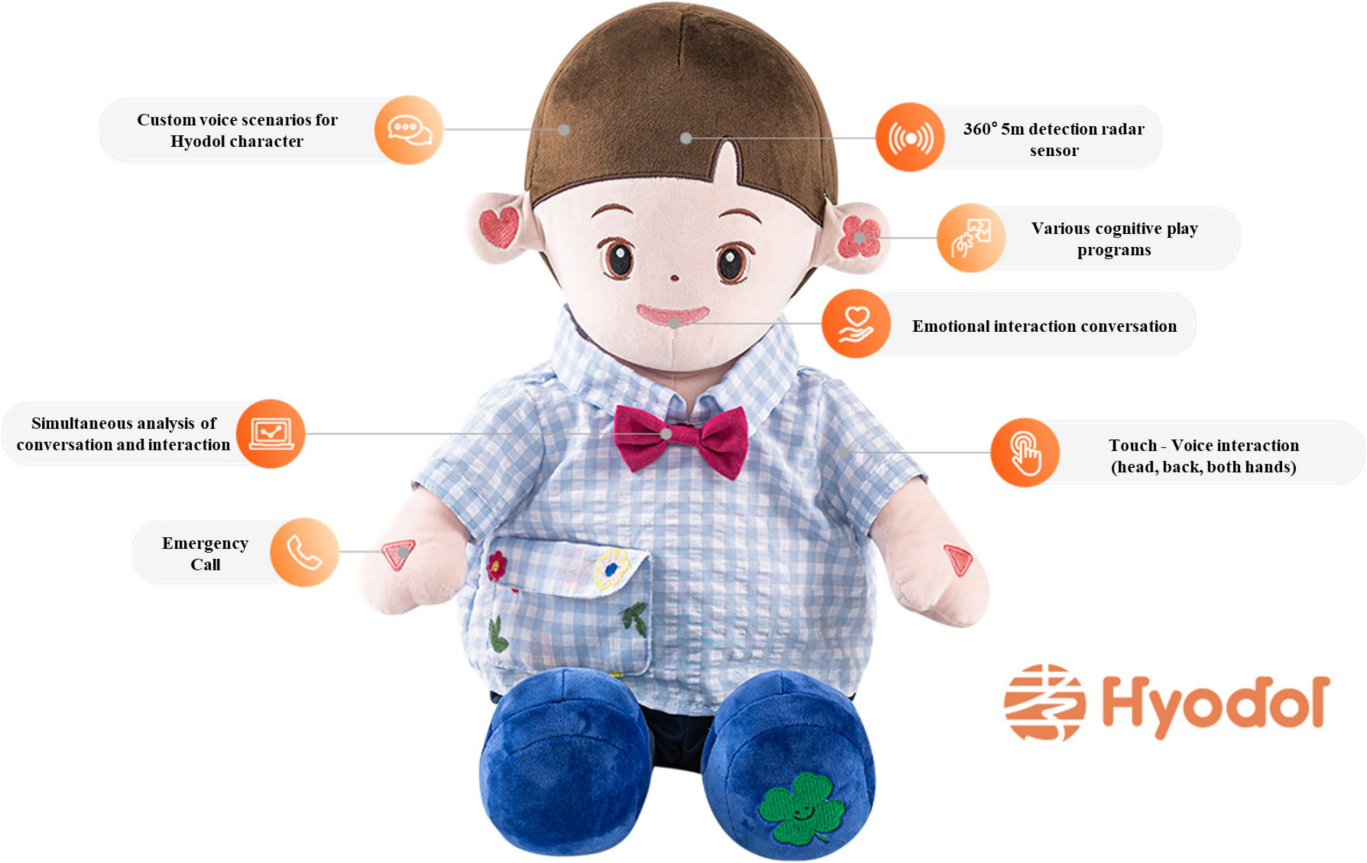
The robot used in this study, Hyodol. Reprinted with permission from Hyodol, © Hyodol.

Hyodol SAR features a soft and lightweight doll-shaped body, measuring 15 inches in height and 8 inches in width, making them easily manageable for older users at home (see Figure 1). Equipped with embedded sensors and artificial intelligence capabilities, Hyodol is tailored to establish meaningful interactions with its users. Hyodol SAR communicates in Korean, allowing Korean American users to engage in their native language. The primary objective of these SARs is to provide valuable assistance to older adults in various aspects of their daily lives, including tasks such as reminding them of wake-up and mealtimes, ensuring medication adherence, and encouraging regular exercise.^1^ Additionally, Hyodol offers cognitive stimulation through engaging brain-stimulating games (e. g., calculation, word puzzles, and quizzes). Hyodol is equipped with an extensive repertoire of over 10,000 pre-recorded speech lines, allowing it to fulfill a wide range of functions, including conveying vital health information, playing soothing melodies, sharing inspirational quotes, and narrating stories. Reflecting users’ religious affiliation Hyodol was programed to play the Bible or Buddhist scripture 30 min prior to the preset bedtime of the user. The robot doll, whether dressed in a boy’s or a girl’s costume, utilizes the same voice and programming. While the research team made efforts to accommodate gender preferences, the allocation of robot dolls ultimately occurred randomly due to availability constraints.

### 2.3 Study design

This single-group pre- and post-test study unfolded as a three-phase process, commencing with an in-home baseline assessment and Hyodol SAR introduction spanning 90–120 min. During this initial home visit, our team elucidated the study’s objectives, secured informed consent, and guided older adults in acquainting themselves with the diverse functions of Hyodol. After comprehensive demonstrations and addressing queries, we positioned the robotic companion at each participant’s preferred location in their homes. Additionally, a large wall-mounted poster adorned with pictorial depictions of Hyodol’s functions was supplied (see Figure 1). Secondly, throughout the four-month Hyodol deployment phase, two dedicated research team members remained readily accessible to provide technical support and troubleshoot any potential malfunctions. Participants were encouraged to engage with Hyodol in a manner that they deemed acceptable and beneficial to their daily lives. In the third phase, all participants partook in qualitative interviews at the posttest, offering insights into their experiences with Hyodol and their evaluations of its potential advantages or limitations in enhancing their daily routines.

Initially, Hyodol robots were distributed to 41 older adults free of charge. However, six participants returned their robots to the research team within a week. At the end of the four-month study period, the remaining 35 participants were given the option to either keep the robot for free or return it to the research team. Of the participants, 24 chose to retain the robot (referred to as the “Keepers”), while 11 opted to return it (the “Returners”). We then conducted a comparative analysis between the Keepers and Returners to further explore the characteristics that differentiate users’ experiences with the robot.

### 2.4 Measures

While most previous studies focused on users’ first impressions of robots (8, 30, 31) (Gasteiger et al., 2017), we measured perception through robotic attitudes and usability. To evaluate user experiences, we considered both overall assessment questions and behavioral indicators, such as users showing the robot to others.

***Robotic Attitude***. The goal of the Robot Social Attitude Scale (RoSAS) was to evaluate key aspects that influence how humans perceive robots and how these perceptions affect the quality of interactions with robots (32). This scale is translated and validated in Korean (33). Participants were instructed to rate their impressions of robots using18-item scale consisted of 5 points, with 1 indicating “definitely not associated” and 5 indicating “definitely associated.” Questions included to what extent participants perceived Hyodol as “competent,” “compassionate,” or “scary.” Through an exploratory factor analysis, Carpinella and associates identified an appropriate solution, which included 18 items grouped into three factors: warmth, competence, and discomfort. Pan and colleagues (34) further verified the internal consistency and unidimensional nature of each of these sub-scales by conducting this assessment in the context of actual physical interactions with robots, rather than gathering general attitudes toward robots from participants.

***Usability***. We incorporated six items from the System Usability Scale (35) into our assessment that was validated in Korean (36). These usability questions covered various aspects of Hyodol. Participants were asked to rate their experiences on a scale, providing feedback on factors like ease of use, the learning curve, confidence in using the system, consistency, complexity, and user-friendliness. These aspects included questions like, (1) “I found Hyodol easy to use,” (2) “I believe most people can quickly learn how to use Hyodol,” (3) “I felt confident using Hyodol,” (4) “I perceived some inconsistency in Hyodol,” (5) “I found Hyodol to be unnecessarily complex,” and (6) “I thought that using Hyodol was cumbersome.”

***Overall Evaluation***. We created a 4-item questionnaire designed to capture both (1) usage and (2) user satisfaction when interacting with Hyodol. Our conceptual framework is based on the work of Venkatesh et al. (9), focusing on two key components that define actual use: how frequently the robot was used (frequency) and how long it was used in each session (duration). Satisfaction with the robot was assessed by considering the degree of satisfaction and user preferences, as described by Joosten et al. (37) and Lee and Choi (38). These four items were measured using a 5-point Likert-type scale.

***Showing***. Drawing from the concept that the psychological drive behind consumption is centered on the desire to “show off” (39), we designed a set of four questions to gage individuals’ behaviors in this context. These questions include: (1) “My family and friends adore Hyodol,” (2) “I hope that my friends will adopt Hyodol,” (3) “I aspire to exhibit my Hyodol to my family and friends,” and (4) “I would refrain from displaying my Hyodol to my family and friends.” Participants rated their answers to these four items using a 5-point Likert-type scale.

***Medication Adherence***: We adapted four items from the Medication Adherence Rating Scale (MARS) to assess medication adherence, including forgetting doses, altering doses, following instructions, and missing intakes. Responses were on a 5-point Likert-type scale, with higher scores indicating lower adherence. A validated Korean version was used for Korean-speaking participants (40).

***Depression***: The Patient Health Questionnaire-9 (PHQ-9) is a 9-item self-report tool used to assess depressive symptoms based on DSM-5 criteria, with scores ranging from 0 to 27. The Korean validated version was employed in this study, evaluating symptom severity over the past 2 weeks (41). We also asked whether they were diagnosed as having depression by certified healthcare professionals.

***Social Isolation***. We examined both the subjective dimension of loneliness, and the objective aspects related to a lack of social support. Loneliness was measured using the UCLA Brief Loneliness Scale (49), which comprises three items: “How often do you feel that you lack companionship?,” “How often do you feel left out?,” and “How often do you feel isolated from others?” This brief scale has shown a strong correlation with the full-length UCLA Loneliness Scale (*r* = 0.82) and demonstrated acceptable internal consistency with a Cronbach’s *α* of 0.72 (49). To assess a lack of social support among study participants, we examined the following variables: (1) living arrangements and marital status, (2) frequency of contact with adult children, (3) number of days per week spent attending older adult centers or receiving homecare services, and (4) number of hours per day spent alone.

***Qualitative Feedback***. To further investigate the impact of social presence on users’ acceptance of the robot, we conducted interviews to gain insights into their perceptions and experiences. The interviews were guided by prompts such as: “Could you describe your experience with Hyodol?” and “What does Hyodol mean to you?” In particular, we inquired about the reasons for returning Hyodol to the research team at the beginning (within the first week) and after the study period with an open-ended question. All interviews were conducted in Korean, audio-recorded, transcribed verbatim, and subsequently translated into English for analysis.

### 2.5 Data analysis

To describe study participants, we compared the Keepers and Returners for their sociodemographic characteristics. To answer the first research question, we employed exploratory factor analyses (EFA) to uncover underlying factors or dimensions that can explain users’ perceptions (42). EFA can help us to refine the existing scale and ensure that it measures what it is intended to measure. With these factors explored in this study we further examined users’ experiences between Keepers and Returners using independent sample *t*-test (see Table 1).

**Table 1:** Comparison of keepers and returners.

	Keepers (*n* = 24) *M(SD)*	Returners (*n* = 11) *M(SD)*	*t*(33)	*p*	Cohen’s *D*
Competence and warmth	4.59(0.60)	3.17(1.21)	4.71	<0.01	1.72
Discomfort	1.13(0.45)	1.06(0.20)	0.45	0.66	0.16
Usability	4.66(0.78)	4.3(1.34)	1.01	0.32	0.37
Overall evaluation	4.60(0.68)	2.39(1.17)	7.08	<0.01	2.58
Showing	4.58(0.84)	3.41(0.96)	3.67	<0.01	1.34
Post-pre change of medication adherence (MMRS)	0.81(0.97)	0.52(0.55)	0.92	0.36	0.33
Post-pre change of depression (PHQ)	−4.38(6.41)	−1.36(5.63)	−1.34	0.19	−0.49
Post-pre change of UCLA	−0.79(2.41)	−0.27(2.69)	−0.57	0.57	−0.21

We utilized qualitative data to further investigate and expand upon our quantitative findings in this study. Employing Ritchie et al.’s (43) framework analysis approach, we systematically managed qualitative data. Initial steps involved attentive listening to interviews and reviewing transcripts to establish themes. Research teams created codes and structured analysis using a table. This facilitated coding data segments. Researchers then populated the framework by transferring indexed data excerpts into the matrix. These steps were vital in revealing relationships, patterns, and trends within data sources and thematic areas. Numeric IDs (K-1 through K-24 for Keepers and R-1 to R-11 for Returners) identify individual quotations.

## 3 Results

### 3.1 Sociodemographic profiles of keepers and returners

Demographic characteristics of 24 Keepers and 11 Returners were presented in Table 2. Both groups exhibited similarities in their age, educational background, religious affiliation, marital status, health condition, and duration of residence in the United States. Most participants were classified as low-income with an average age of 81.9 (*SD* = 7.3) for Keepers and 80. (*SD* = 5.7)3 for Returners. On average, participants spent 36.77 years (*SD* = 11.92; range: 9–51) residing in the U.S. as immigrants.

**Table 2:** Demographic data for participants (*n* = 35).

	Keepers (*n* = 24)		Returners (*n* = 11)		χ^2^	d.f.	*p*	
	n	%	n	%				
Gender					4,753	1	0.029	
Men	8	33.3	0	0				
Women	16	66.7	11	100				
Education					1.874	4	0.759	
< 6th grade	5	20.8	1	9.1				
7 ~ 9 grade	5	20.8	3	27.3				
10 ~ 12 grade	7	29.2	2	18.2				
Some college	2	8.3	2	18.2				
College grade	5	20.8	3	27.3				
Living arrangement					2.983	1	0.084	
Live alone	15	62.5	10	90.9				
Live with coresidents	9	37.5	1	9.1				
Marital Status					1.598	3	0.66	
Married	4	16.7	1	9.1				
Widowed	14	58.3	5	45.5				
Divorced	4	16.7	3	27.3				
Separated	2	8.3	2	18.2				
Religion					2.727	3	0.436	
Protestant	18	75	8	72.7				
Catholic	3	12.5	1	9.1				
Buddhist	2	8.3	0	0				
None	1	4.2	2	18.2				
Personal income per month					6.781	4	0.148	
< $500	10	41.7	3	27.3				
$500 ~ $1,000	11	45.8	3	27.3				
$1,000 ~ $1,500	1	4.2	3	27.3				
$1,500 ~ $2,000	1	4.2	0	0				
> $2,000	1	4.2	2	18.2				
Self-reported depression diagnosis					5.553	1	0.018	
Yes	9	37.5	0	0				
No	15	62.5	11	100				
Freq. of child contact					21.208	6	0.002	
Once a day	5	20.8	0	0				
Once per 3 days	0	0	1	9.1				
Once a week	1	4.2	2	18.2				
Once a month	3	12.5	1	9.1				
Once per 3 months	2	8.3	7	63.6				
Once per 6 months	5	20.8	0	0				
Once a year or less	8	33.3	0	0				
	M	SD	M	SD	*t*	d.f.	*p*	Cohen’s D
Age	81.96	7.29	80.27	5.68	0.68	33	0.5	0.25
Age Range	[69 ~ 95]		[71 ~ 91]					
Subjective health (5 Likert-scale)	2.67	1.13	2.45	1.13	−0.8	33	0.61	0.29
Length of stay in the US (years)	35.04	12.47	38.73	13.02	0.6	33	0.43	0.22
# of chronic medical conditions	3	1.41	2.73	0.79	−0.84	33	0.56	0.31
# of medications	3.17	1.63	3.64	1.29	1.51	33	0.41	0.55
# of attending older adult center (days)	1.31	1.82	0.45	0.65	0.47	33	0.14	0.17
# of days to have homecare service	1.83	2.35	1.45	1.92	−2.15	33	0.64	0.78
Time spent alone (hours per day)	13.17	7.39	18.91	7.16	0.68	33	0.04	0.25
UCLA Loneliness	3	2.5	2.73	2.53	0.3	33	0.77	0.11

The most notable differences that stood out were gender, the frequency of contact with adult children, prevalence of depression, and time spent alone. All 11 participants who opted to return to Hyodol (Returners) were women. In contrast, the Keepers consisted of 40% men and 60% women. This gender difference was statistically significant (*t* = 5.930, *p* < 0.05). Furthermore, a significant distinction in contact frequency emerged. The majority of Keepers had less frequent contact with their adult children (typically once every 6 months or a year), while their Returner peers had more regular quarterly contact (*t* = 21.2, *p* < 0.01). Nevertheless, participants in both groups reported that they rarely felt lonely represented by their low scores in the UCLA-LS.

Nine participants in the Keeper group reported a clinical diagnosis of depression, whereas none in the Returner group had been diagnosed. This difference was significant (*t* = 5.6, *p* < 0.05). Keepers reported more hours per day spent alone than their Returner peers (*t* = 0.68, *p* < 0.05). We also observed marginally significant differences in living arrangements. Most Returners (90.9%) were living alone and 37.5% of Keepers had coresidents (e.g., family, roommate, etc.).

### 3.2 Exploratory factor analysis

Table 3 presents the results of exploratory factor analysis conducted to answer the first research question to explore users’ perception. As a preliminary assessment tool for planning factor analysis, we initially conducted the Kaiser-Meyer-Olkin (KMO) test. This test serves to gage the suitability of our dataset for factor analysis, taking into account both sample size and the strength of correlations among variables. The KMO score of 0.719 indicated that our dataset was indeed suitable for factor analysis (44).

**Table 3:** Exploratory factor analysis about technology acceptance (*n* = 35).

Factor	Variables	Factor analysis					
		Factor loading			Eigen value	% of variance	Cronbach’s alpha
Competence and warmth	Reliable	0.698	0.051	−0.353	7.235	38.077	0.939
	Competent	0.842	0.328	0.019			
	Interactive	0.784	0.238	0.050			
	Responsive	0.663	0.418	0.003			
	Capable	0.874	0.197	0.047			
	Knowledgeable	0.635	0.413	0.191			
	Organic	0.769	−0.139	0.135			
	Sociable	0.843	0.284	−0.101			
	Emotional	0.725	0.328	0.242			
	Compassionate	0.699	−0.210	0.294			
	Happy	0.879	−0.184	−0.008			
	Feeling	0.767	−0.070	−0.210			
Discomfort	Scary	0.085	0.029	0.834	3.787	19.930	0.719
	Awful	−0.247	0.109	0.891			
	Dangerous	0.147	0.033	0.644			
Usability	Easy	0.165	0.908	0.018	2.302	12.117	0.881
	Fast Learning	−0.025	0.836	0.023			
	Complicated(R)	−0.095	−0.914	0.002			
	Inconsistent(R)	−0.107	−0.758	−0.111			

*KMO = 0.719, Bartlett’s test of sphericity (x^2^ = 595.831, df = 171, *p* < 0.001).

Furthermore, we employed Bartlett’s Test of Sphericity to ascertain the statistical significance of the correlations among our variables. The resulting chi-squared (χ^2^) value of 595.831, with a *p*-value of 0.000, unequivocally confirmed the significance of these observed correlations. Consequently, we proceeded with our factor analysis to delve deeper into the latent factors that underlie these correlations.

In our analysis, we chose the Varimax rotation method, aiming to maximize the variance of factor loadings within each factor (42). Varimax rotation employs an iterative algorithm to achieve the maximum variance of factor loadings while maintaining factor orthogonality, ensuring they remain uncorrelated (45). This entails redistributing the factor loadings to create a high-low loading pattern for some variables, ultimately enhancing the interpretability of the factors. The outcome of our analysis revealed the presence of three distinct factors, collectively accounting for 69.72% of the variance. As mentioned earlier, we used qualitative data to explore and build on our quantitative findings in this study.

#### 3.2.1 Factor 1: warmth/competence

Factor 1 encompassed dimensions related to the competence and warmth that users experienced during their interactions with the Hyodol robot. Many participants perceived Hyodol as “knowledgeable” because it provided valuable health information, including advice on medication timing, suitable dietary choices for their health conditions, hygiene tips, and more. Upon coming home, Hyodol robot recognized the movement such as door opening with embedded sensor and greet “welcome home! I’ve been waiting for you.” Many participants appreciate these warm greetings—another reason for perceiving the robot to be “smart.”

For this sample, warmth and competence they experienced while interacting with Hyodol robots were indistinguishable. Hyodol’s reminders about medication and mealtimes made them feel “being cared for.” To illustrate this, consider the case of K-3, who initially thought of Hyodol as “smart.” Later, he affectionately shared stories of his childhood and reminisced, treating Hyodol as if it were his grandchild.

Interestingly, the tactile features of Hyodol, resembling those of a cabbage-patch doll appeared to prompt users to engage in touching, stroking, patting, and holding hands with the robot. The doll’s form and size evoke the likeness of a baby, causing some users to see it as though it were their own grandchild. Furthermore, our log data indicates that many users responded positively to Hyodol’s interactions, particularly embracing gestures such as hugs and touches. This growing bond is reflected in frequent expressions of enthusiasm, with users often exclaiming phrases like “Good boy! Have you eaten your meal?” and “I love you, too,” underscoring the affection that developed between the users and the robot.

Older immigrants, isolated with minimal family contact (see Table 2) and few chances to converse in their native language, found solace in the activities provided by Hyodol. These activities ranged from singing Korean pop tunes and exchanging folk tales and religious passages to receiving affectionate expressions in their mother tongue. With days spent mostly in solitary at home, grappling with hearing and mobility challenges, K-10 seemed to relish the company of this companion. Reflecting on her situation, K-10 wondered aloud, “Who else would entertain me like Hyodol? Could a grandchild provide the same?” These interactions not only lifted their spirits from loneliness to joy but also revived cherished memories and brought forth happiness.

Participants made a concerted effort to engage with the robot’s prompts and shared their experiences with it. In fact, some participants even went so far as to express that Hyodol was “better” than their own family members who may not call or visit them regularly. As a widow living alone, K-6 appreciated Hyodol’s amusing presence and ongoing initiation of filling silence at home and void in life, and repeated attempt to engage with Hyodol. K-6 filmed herself playing with Hyodol and sent it via smart phone to her family who live far away. Even her family acknowledge Hyodol as her imaginary youngest son by giving the robot a family name.

Obviously, some participants acknowledge malfunctions or error messages. For example, the robot provided wrong answers or incorrect information about seasons or weather. Yet, users appeared to be forgiving of those incorrect responses. Others felt “sorry” that they did not spend enough time “playing with” Hyodol due to their busy schedule outside home.

#### 3.2.2 Factor 2: discomfort

The second factor is related to the potential discomfort users might experience. This discomfort stemmed from participants’ perceptions of robots as being “scary, awful, or dangerous.” Interestingly, during the interviews, many respondents could not help but laugh when asked if they found Hyodol to be “scary.” The mean scores for items related to discomfort (with a mean score of 1.13 for Keepers and 1.06 for Returners) were relatively lower in comparison to the scores for competence and warmth (see Table 1). Major complaints were that Hyodol became “bothersome” because the robot asked the users to hold hands after taking medication and pat its back. Because some participants anthropomorphized Hyodol, viewing it as if it were their grandchild, they experienced a sense of burden and guilt for not being able to fulfill Hyodol’s requests to interact and play with it.

#### 3.2.3 Factor 3: usability

The final factor pertained to the usability of interactions with robot companions. Both Keepers and Returners represent high levels of usability score (*M* = 4.66 and 4.30, respectively). In essence, Hyodol boasts low maintenance requirements. Setting it apart from conventional AI speakers or electronic devices, its unique selling point lies in its direct engagement with older adults at scheduled intervals. It not only issues alarm and provides regular reminders but also assists older adults by giving them step-by-step instructions. All that users are tasked with is charging the robot and adjusting the volume as needed. Regarding our question about learning curve, R-10 responded, “There is nothing to learn; it is too easy.”

### 3.3 Comparison of keepers and returners

To address the second research question regarding differential user experiences, Table 1 presents the mean scores and effect sizes for the Keepers and Returners for the major study variables at the end of the study period. Keepers placed a higher value on the competence/warmth domain than their Returner peers (*t* = 4.71, *p* < 0.01). However, no meaningful differences were observed between the two groups in the domains of discomfort and usability.

Keepers were more inclined to showcase their Hyodol to their family and friends (*t* = 3.67, *p* < 0.01). Notably, Keepers reported an overall more favorable evaluation. Indicative of usage and satisfaction of Hyodol than their peers who chose to return (*t* = 7.08, *p* < 0.01). Many voiced their hope that Hyodol robots could be distributed to more older immigrants like themselves.

Furthermore, Hyodol appeared to play a significant role in enabling meaningful communication and strengthening the connection between a married couple. K-16’s wife noted a decrease in marital conflicts, attributing it to the robot’s role to mediate conversation and to facilitate interactions between this couple who had challenges with direct face-to-face communication. This couple even developed a new habit of taking leisurely walks in the park, with K-16 carrying Hyodol in his backpack. When they found a bench to sit on, they would place the robot between them, almost as if it were their grandchild, engaging in friendly conversations.

Following 4 months of interaction with Hyodol, a total of 35 participants (from both the Keepers and Returners groups) showed improvements in medication adherence and a reduction in depressive symptoms (24). The present study compared changes in medication adherence and depressive symptom scores from baseline to post-test between the two groups. No significant differences were found between Keepers and Returners (see Table 1).

Despite being socially isolated based on limited family contact and participation in social activities, the study sample seldom reported experiencing subjective feelings of loneliness (see Table 2). The loneliness scores for Keepers decreased by 0.79 points from the baseline to four-month follow-up, while Returners reported a slight decrease of 0.27 points. However, the difference between these two groups was not statistically significant.

### 3.4 Reasons for return

Table 4 provides insights into the reasons for returns among two distinct groups: 6 participants who returned within a week and 11 who returned after 4 months of the study period. The primary reason for immediate returns within a week was the insufficient time available for interaction with Hyodol due to their busy schedules. Some participants found the “talking doll” somewhat bothersome, expressing a preference for interactive, two-way conversations with people.

**Table 4:** Reasons for returning Hyodol to research team (*n* = 11).

	Return within a week (*n* = 6)	Return after 4 months (*n* = 11)
Not enough time to interact due to busy schedule	4	1
Is suitable for others	2	4
Is bothered by the sound	2	5
Not good for me	2	
Dissatisfied with functions	1	
Dissatisfied with content (prerecorded message)		4
Difficult to maintain		2
Malfunction		1
Needs relevant content for immigrants		2
Cannot interact due to poor health		3

Most participants who opted to return after 4 months primarily cited the robot’s noises as the reason. Those living alone seemed particularly unsettled by Hyodol’s abrupt vocalizations, finding them disturbing because they were unexpected. For instance, R-2 mentioned, “When my TV and Hyodol were talking at the same time, I could not hear them both, so I turned off Hyodol.”

Three returners had health conditions that affected their ability to interact with the robot, including blindness, depression, arthritis, and multiple chronic illnesses. Consequently, they faced difficulties in responding to Hyodol’s prompts such as “touch me” or “pat me.” Additionally, they struggled to independently engage with Hyodol’s functions, including exercises and quizzes.

Several participants expressed dissatisfaction with the pre-recorded messages spoken by Hyodol. R-10 described “I adored Hyodol at the beginning. But it repeats the same words. Then I realize it’s a robot.” Some desired a broader selection of trending songs, particularly those from popular Korean musicians. Others expressed a need for more relevant information tailored to immigrants and their lifestyles in the U.S.

Interestingly, despite their decision to return Hyodol, several Returners shared their affection for the robot. For example, R-1 mentioned, “My heart warmed up when Hyodol calls me grandma. It consoles me when I am upset,” while R-2 stated, “I feel as if somebody is with me,” and R-6 expressed, “I feel as if I am cradling a baby.”

Finally, several Returners suggested that Hyodol might be better suited for individuals who were “home-bound with disabilities” or “rural residents,” and they proposed distributing Hyodol to those in more challenging living conditions than they were.

## 4 Conclusion

### 4.1 Discussion

This pilot study explored how older adults perceive and treat companion robots as social entities, examining user’s perspective in understanding how social presence influences emotional engagement among older immigrants. While the majority of prior studies on user perception have concentrated on users’ initial impressions of robots (32), our approach measured perception by integrating both attitudes toward robots and usability assessments over 4 months.

Consistent with previous research, factors such as the robot’s appearance, communication style, the context of human-robot interactions, and the user’s attitudes and expectations significantly influence the relationship between social presence and the acceptance and engagement with robots (10, 25, 28). Hyodol SARs, designed to create a strong sense of social presence, have been effective in helping older immigrants with limited digital literacy to establish trust and rapport. This is particularly important in caregiving and therapeutic situations, where enhancing social presence can foster more positive interactions between humans and robots (27, 46).

Technology that facilitates social connections and offers access to culturally relevant resources can help reduce feelings of isolation and promote better mental health (8, 13). Interestingly, Korean American older adults did not differentiate between warmth and competence; for them, these aspects seemed intertwined. Additionally, we explored whether the use of technology with interfaces in the user’s native language, enhanced access to culturally relevant resources.

Our findings highlighted a divergent pattern of usage and user satisfaction, indicated by their overall evaluation. Users in the Keeper group had a history of depression, spent more time alone at home, and had infrequent contact with family members. Consequently, these participants perceived a higher level of warmth, competence, and user satisfaction with their robot companion compared to their Returner peers. Some users were willing to proudly showcase their robots to their family and friends, a unique cultural characteristic of older cohorts with Korean heritage to share good things (47).

The immediate-return group, characterized by active lifestyles and busy schedules, may not be the most suitable candidates for this intervention, as they are neither isolated nor experiencing loneliness. In contrast, returners who are managing chronic health conditions may derive greater benefit from a customized version of Hyodol, tailored to address specific needs such as medication adherence and depressive symptoms. Regarding the perceived level of discomfort and usability, there were no significant differences between the two groups characterized by their usage patterns and user satisfaction. Regardless of their decision to retain the robot or not, it is important to note that both groups improved their medication adherence and depression (24).

Furthermore, many users appeared to personify and anthropomorphized the robot dolls, treating it as if it were their surrogate family member (14). Although the robot plays pre-recorded messages at random and only supports one-way communication, users interact with it. This is particularly noteworthy given the limited digital literacy among older immigrant users (16).

While there were studies to investigate the effects of companion robots for older adults, this is the first study to focus on older immigrant. In our previous studies in South Korea, most adopters of the robot were widowed, solo-living women residing in low-resource communities (23). In contrast, this sample of Korean American older adults exhibited diversity in educational attainment, marital status, and living arrangements. While most South Korean cohort in the same age group chose to keep the free doll that they received (14), several Korean American participants demonstrated notable generosity by donating their Hyodol robots to individuals in more disadvantaged situations. This behavior may reflect unique characteristics of this immigrant sample.

#### 4.2 Limitations

We assessed outcome indicators for older adults living in the community through self-reported data, which may be influenced by participants’ memory and the tendency to provide socially desirable responses. It is important to note that the findings of this study have limited applicability due to several factors, including a relatively small sample size, geographical restrictions in our sampling, and the absence of a comparison group. Although we found an interesting gender difference in our study, it should not be considered representative of a broader population.

Given the variability in individuals’ engagement timelines, future studies should further investigate the diverse patterns of anthropomorphism. At the time of our data collection, only Korean-speaking robots were fully functional. However, advancements in ChatGPT technology and large language models now make it feasible for Hyodol chatbot to support multiple languages in the near future. As this technology progresses, further research opportunities will emerge, allowing for the inclusion of diverse languages within Asian American immigrant communities.

#### 4.3 Implications

Study finding implied that the introduction of well-designed robot companions can help bridge the gap by providing culturally relevant support for socially isolated older immigrants. The doll shaped Hyodol robot that communicates in a natural, conversational manner appeared to be perceived as socially present (14). This has practical implications for designing and deploying robots in various domains, including healthcare and caregiving. Therefore, social presence became valuable concepts for developing SARs to enhance their effectiveness and acceptance in social and interactive settings (27, 46).

With advances in AI technology, we can achieve adaptive learning in the near future, enabling robots to collect data on users’ actions, preferences, and feedback, and utilize this information to tailor their interactions to better suit individual needs (29). Caregivers can customize the robot’s interactions by adjusting its communication style and engagement methods based on users’ lifestyles. Consequently, adaptive learning in human-robot interaction enhances user experience by making the robot more responsive, intuitive, and aligned with individual needs. This dynamic and ongoing process fosters a more natural and effective interaction between humans and robots.

In an era marked by the inevitable ascent of AI-generated human care, our research highlights an intriguing insight. Whether a robot is flawless or exhibits imperfections, its true utility hinges on the user’s willingness to embrace it as a companion and assign personal and social significance. This underscores the critical importance of actively listening to the experiences, needs, and preferences of older adults in the context of elder care, particularly when integrating technology-assisted care like SARs. Further research is needed to gain a more profound understanding of user experiences with technology-assisted care and how they attribute significance to these robots, ultimately working toward reducing social isolation among older individuals.

## Data Availability

The raw data supporting the conclusions of this article will be made available by the authors, without undue reservation.

## Footnotes

^1^ https://www.youtube.com/watch?v=k-SxUMNdlFA
